# Developmental Coordination Disorder before the Age of Three: A Longitudinal Retrospective Study in a Belgian Center for Developmental Disabilities

**DOI:** 10.3390/children9030334

**Published:** 2022-03-02

**Authors:** Nina Vens, Griet Dewitte, Hilde Van Waelvelde, Lynn Bar-On, Amy De Roubaix

**Affiliations:** 1 Rehabilitation Sciences, Ghent University, 9000 Ghent, Belgium; grietje.dewitte@ugent.be (G.D.); hilde.vanwaelvelde@ugent.be (H.V.W.); lynn.bar-on@ugent.be (L.B.-O.); amy.deroubaix@ugent.be (A.D.R.); 2 Center of Developmental Disabilities, 9000 Ghent, Belgium

**Keywords:** developmental coordination disorder, movement quality, motor test, early diagnosis

## Abstract

This study aimed to explore the association between developmental coordination disorder (DCD) diagnosed after the age of three and both a standardized motor test—the Alberta Infant Motor Scale (AIMS)—and non-standardized observation of movement quality carried out before the age of three. Children at risk or with developmental concerns were studied retrospectively. Children were excluded in case of a diagnosis, excluding DCD, e.g., cerebral palsy, or IQ < 70. Of the 503 included children, 246 were diagnosed with (at-risk) DCD. Multivariate binary logistic regression revealed a significant association between DCD diagnosis after the age of three and male gender and with different aspects of poor movement quality in different age groups before the age three. Univariate analyses revealed an association between DCD diagnosis and the number of poor movement-quality descriptions at 0–6 months, 6–12 months, and 18 months–3 years but not with the AIMS scores. The MABC-2 scores after the age of three were significantly correlated with the number of poor movement-quality descriptions in age groups 0–6 months and 18 months–3 years and with the AIMS scores in age groups 6–12 months and 12–18 months. The results suggest that DCD can be associated with poor movement quality before the age of three.

## 1. Introduction

Developmental coordination disorder (DCD) is a neurodevelopmental disorder, a lifelong condition that makes it hard to learn motor skills and coordination. The Diagnostic and Statistical Manual of Mental Disorders 5th edition (DSM-5) outlines four diagnostic criteria: (A) the acquisition and execution of motor coordination skills are substantially below age expectations despite ample opportunities to learn; (B) these deficits significantly interfere with the performance of activities of daily living and impact academic productivity, leisure, and/or play; (C) the onset of symptoms is in the early developmental period; and (D) the motor skills deficit is not better explained by intellectual disability, visual impairment, or a neurological/medical condition affecting movement [1]. DCD is usually not an isolated disorder of motor functioning but a complex condition frequently associated with other neurodevelopmental disabilities, such as attention deficit hyperactivity disorder (ADHD), learning disabilities, speech and language impairment, and autism spectrum disorder (ASD). Despite DCD being one of the most common childhood neurodevelopmental disorders, affecting 1.8 to 6% of school-aged children [2], it is often misunderstood and frequently not diagnosed until school-age if at all [3]. Previous guidelines of the European Academy of Childhood Disability (EACD) recommended that formal DCD diagnosis should not be made before the age of three [4]. The latest revision of these guidelines no longer explicitly excludes a diagnosis before the age of three but recommends limiting the diagnosis before the age of five to severe cases. In such instances, the decision to make a diagnosis should be based on the findings from at least two motor assessments carried out at least three months apart. They point to the lack of stability of the DCD diagnosis at early ages [5]. Subsequently, early diagnosis is uncommon, and research regarding the motor development of children with DCD before the age of three is scarce. However, early identification of developmental disorders is crucial to provide a basis for an appropriate educational and treatment program, to enhance quality of life of the child and parents, and to prevent or minimize physical, emotional, and behavioral consequences [5,6,7].

An important issue in the discussion on early diagnosis of DCD is the diagnostic criteria. The EACD guidelines [5] suggest that criterion A be satisfied by using the Movement Assessment Battery for Children second edition (MABC-2) or the Bruininks–Oseretsky Test of Motor Proficiency (BOT-2) although these tests can only be used from, respectively, the age of three and four years. In the absence of generally accepted cut-offs for identifying DCD, it is recommended that when using the MABC-2 or other equivalent objective measures, the 16th centile (1 SD) for the total score should be used as a cut-off. Scores at or below the fifth centile should be considered as unequivocal evidence of DCD provided the child meets all other criteria. However, these guidelines are not specifically established with a younger population in mind.

The EACD guidelines also inform on the limitations of the MABC-2. The specificity seems to be good (0.8–0.9), but the sensitivity (0.7–0.8) is generally lower [5]. In addition, in the majority of studies, the reliability of MABC-2 was studied in typically developing children and not in clinical populations [8,9]. For example, a recent study on the reliability of the MABC-2 in preschool children excluded children with a medical diagnosis, children who refused to perform on the retest, or children who scored below the fifth centile on one test and above the 16th centile on the other test (*n* = 9/183) [10]. The authors assumed that such a shift from one category to another was a motivational and not an ability issue. However, such shifts are not unusual in the clinic. Children, especially young children, are not always cooperative. They may be sick, tired, or unable to perform in unfamiliar contexts. Clinicians observe these circumstances and, rather than dismissing the results, may take them into account when considering the diagnosis of DCD.

Clinical practice in Belgium reveals that the MABC-2 cut-off is only indicative but not absolute to evaluate criterion A. The test performance may be under- or overestimated. Some children perform quite well on the MABC-2, such as when the test is administered in a quiet environment that allows the child to concentrate on the motor activity. These children fail only when environmental stress increases, when the task is complex, or when it is a double task. If the history and checklists of the parents and the school clearly show a strong impact of motor problems on daily life (criterion B), and the non-standardized observations of the child’s motor performance confirm these problems, clinicians in Belgium will consider the diagnosis of DCD even with a MABC-2 score above the 16th percentile. Since there are no formally approved guidelines for the diagnosis of DCD in Belgium, clinicians do not have a clear cut-off score to exclude the diagnosis of DCD. Rather, regular meetings of the different Belgian Centers for Developmental Disabilities on the application of the DSM-5 criteria and the EACD guidelines provide a common reference frame to support the multidisciplinary teams in their clinical diagnostic decisions. The EACD also draws attention to the distinction between clinical diagnostic criteria and research criteria [5]. For clinicians, it is important not to miss children in need of adequate support. Therefore, in the Belgian Centers for Developmental Disabilities, the MABC-2 results are only indicative, and together with multidisciplinary anamnestic information and clinical observations, diagnostic decisions based on the four DSM-5 criteria are made. Internationally, there is no consensus yet on early identification of DCD. However, there is growing recognition that early intervention could be beneficial [7]. Therefore, not only is early identification important but also knowledge about the early symptoms of DCD such that those children who need to be followed up before a formal diagnosis can be given are identified. 

Many studies have been investigating motor outcome of preterm born children although mostly focusing on the prevalence of cerebral palsy (CP). More recently, these studies expanded their focus, revealing that the increasing rate of motor impairment at school age among extreme preterm and/or extreme low-birth-weight children [11] is caused by non-CP-related motor impairment but rather by mild motor impairment or DCD [12]. In this group of preterm-born children, the predictive value of early motor assessment has been frequently investigated. De Roubaix and colleagues reviewed the predictive value of standardized motor assessments before the age of five for school-age motor outcomes excluding CP [13]. They concluded that standardized motor assessments before five years seem valuable in detecting early motor problems. However, no assessment instrument reached 80% sensitivity and specificity. While very few of the reported studies investigated the prediction of a DCD diagnosis, most studies attempt to predict later motor test performance. For example, Spittle and colleagues reported good predictive accuracy of the Alberta Infant Motor Scale (AIMS) scores at 4 months on MABC-2 at 4 years corrected age [14]. Two studies predicted a DCD diagnosis. Kwok et al. concluded that the MABC-2 at 3 years was highly sensitive to predict DCD at 4.5 years in very preterm children but also reported many false positives [15]. Goyen et al. succeeded to predict DCD at school age from the Fine Motor Quotient of the Peabody Developmental Motor Scales—2 (PDMS-2) at the age of three in extremely preterm born children [16]. Additionally, there is evidence for the predictive value of an abnormal General Movements Assessment at fidgety-age (three months) on mild motor impairment at school age although results are not always conclusive [17,18,19]. However, it is remarkable that the scoping review of Lee and Zwicker (2021) on early identification of DCD concluded that no assessment studies have been conducted on term-born infants in relation to later DCD diagnosis [7].

In research, the evaluation of early motor development, e.g., by mean of the Bayley Scales of Infant Development—3 (Bayley-3) or PDMS-2, is based on test items that focus on the product of children’s motor skills, as they are quantitative. How long can the child maintain the sitting position? Can the child stand without support? However, Rosenbaum emphasized the importance of including both qualitative and quantitative observations when making a judgment about a child’s early development [20]. Qualitative observations describe the way things are done and the control and coordination of movement. The AIMS and the Movement Assessment of Infants (MAI) are standardized motor tests that also evaluate qualitative aspects of motor performance. The MAI provides items on muscle tone and primitive reflexes, while the AIMS considers three criteria related to quality of movement: weight distribution, posture, and movement against the force of gravity. Rogers and colleagues pointed out that in an extreme low-birthweight population, the MAI at 8 months corrected age, particularly the volitional movement subtest, more accurately identified infants at greatest risk for DCD at the age of 4 to 5 years compared to the Bayley-3 [21]. Jansen and colleagues aimed to develop a specific qualitative assessment tool, i.e., “Observable Movement Quality”, a measurement tool evaluating 15 well-defined aspects of movement quality [22]. 

In clinical practice, a non-standardized evaluation of movement quality is an important element of the diagnostic process next to standardized motor tests. However, it remains unclear how early movement quality is related to DCD. Clinicians monitoring the motor performance of both preterm and full-term children longitudinally suggest that most children diagnosed with DCD later in life were already described as children with qualitative atypical development. This study aims to explore the association between DCD diagnosed after the age of three and both a standardized motor test—the AIMS—and non-standardized observation of movement quality carried out before the age of three. It is an explorative retrospective study based on longitudinal clinical data from Ghent Center of Developmental Disabilities.

## 2. Materials and Methods

### 2.1. Participants

**Figure 1:**
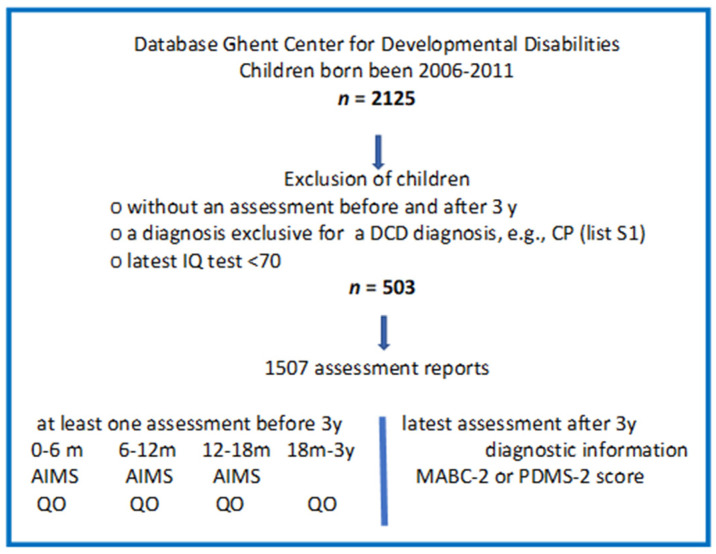
Research flow. m, months; y, years; QO, qualitative motor observation.

All data were retrospectively collected via the database of the Ghent Center of Developmental Disabilities. This center is accredited for multidisciplinary detection, diagnostics, and referral of children with developmental concerns, including follow-up of children with gestational age < 30 weeks and/or birthweight below 1250 g. Assessment reports from all examined children, born between 2006 and 2011, with at least one assessment before and one after the age of three were extracted from the database. Adopted exclusion criteria were (1) diagnoses considered as exclusion criterion for DCD (comprehensive list in Supplementary Materials Table S1) and (2) IQ < 70. Application of these criteria resulted in a data set of 1507 assessments from 503 children. Assessments were categorized in five age groups: 0–6 months, 6–12 months, 12–18 months, 18 months–3 years, and >3 years. When two assessments occurred within one age category, only the assessment at the oldest age was retained. The follow-up assessments of one child were supervised by the same or a different neuro-pediatrician, but the physiotherapist assessing children before the age of three was never the same as the physiotherapist assessing the child after the age of three. The research flow is illustrated in Figure 1. The study was approved by the Medical Ethical Commission of Ghent University Hospital, Ghent, Belgium (B670201730764).

### 2.2. Diagnosis

The (at-risk) DCD diagnosis was extracted from the latest available record. The children were diagnosed following a rigorous multidisciplinary procedure from a team with at least a neuro-pediatrician, a physiotherapist, and a psychologist. DSM-IV or DSM-5 criteria were adopted. To evaluate if motor coordination skills were below the age expectations (criterion A), all children were tested with the MABC-2 except 19 children between three and four years six months, who were tested with the Peabody Developmental Motor Scales—2 (PDMS-2). As recommended by EACD, a cut-off score at, or below, the 16th percentile for children five years and older and at, or below, the 5th percentile for children between three and five years old, was used to label children with an (at-risk) diagnosis of DCD. However, clinical findings could make it necessary to deviate from this general directive when the child was not cooperative or not concentrating during testing or the results were not in line with history and/or observations. In-depth history taking and checklists were used to confirm that the child had enough opportunities to learn motor skills (criterion A), that the deficits significantly interfere with daily life activities (criterion B), and that the onset of the symptoms was in the early developmental period (criterion C). Psychological and medical assessments verified if the motor skills deficit were not better explained by intellectual disability, visual impairment, or a neurological/medical condition (criteria D). For some analyses, at-risk diagnoses were combined with confirmed diagnoses. In these analyses, the combined groups are referred to as the “DCD group”. The group without a (risk of) DCD diagnosis is referred to as the “non-DCD group”.

### 2.3. Standardized Tests

Alberta Infant Motor Scale [23]. The AIMS was designed to monitor motor development in infants at risk of central nervous system dysfunction who might display subtle deviations in performance. It can be used from birth through to 18 months of age or when the infant begins to walk and involves observing the infant in prone, supine, sitting, and standing with minimal handling. Psychometric properties of the AIMS are good [24]. AIMS scores were reported via six percentile (Pc) categories: (1) Pc 1–5, (2) Pc 5–10, (3) Pc 10–15, (4) Pc 15–25, (5) Pc 25–50, and (6) Pc 50–100.

Movement Assessment Battery for Children—second edition [25]. The MABC-2 is one of the most frequently used tests to assess a child’s general motor functioning by means of eight items evaluating manual dexterity, ball skills, and balance with an age range from three to 16 years old. The translated edition in Dutch, norm-referenced in Flanders and the Netherlands, was used. MABC-2 is a reliable and valid measure to assess motor competence in children with DCD [26]. For children aged six years and older, a cut-off score at or below the 16th percentile is recommended for an (at-risk) diagnosis of DCD [4]. MABC-2-scores were reported via standard scores (SS) with mean = 10 and SD = 3.

Peabody Developmental Motor Scales—second edition [27]. The PDMS-2 is a standardized test designed to evaluate both fine and gross motor skills in children from birth to 71 months of age. The PDMS-2 consists of six subtests: reflexes, stationary, locomotion object manipulation, grasping, and visuo-motor integration. The age of the child determined the entry point of the test, and the child had to be able to perform the first three items per subtest correctly. If the child was not able to do so, the subtest was administered backwards until a child reached three consecutive correct scores. Items increase in difficulty, and the test is stopped when children cannot complete items to minimize assessment time. The 16th percentile was considered indicative for the diagnosis of DCD. 

### 2.4. Non-Standardized Data on Movement Quality

Clinical assessment reports from different experienced neuro-pediatricians and pediatric physiotherapists from the Ghent Center of Developmental Disabilities were screened. Firstly, 20 random assessment reports were screened by the first and second author, both physiotherapists at the Center of Developmental Disabilities. Descriptions of poor movement quality were literally copied from the records. Obvious synonyms were merged in consensus with the team of the center and resulted in a list of descriptions. Secondly, these descriptions were meticulously tallied as absent (0) or present (1) in all assessment reports of children < 3 years old. When new terms were found in the reports, the terms were literally added after consultation with the first two authors. Thirdly, during a consensus meeting with the clinicians who produced the reports, similar terms were combined into categories of movement quality. The final comprehensive list of terms and categories is available in Supplementary Materials Table S2. Finally, the number of terms of poor movement-quality descriptions per assessment report were summed into one continuous variable represented as “movement-quality concerns”.

### 2.5. Data Analysis 

Multiple binary logistic regression with manual Wald backward elimination was performed for each age group with the presence of DCD as dependent variable and the categories of movement quality, sex, and prematurity as independent variables. Prematurity is hereby defined as born with gestational age ≤ 36 weeks. In order to reduce the risk of missing important variables, a pre-selection was performed using univariate models. All variables with *p* ≤ 0.25 were withheld for further analysis. This relaxed *p*-value was used to reduce the risk of missing important variables. Independent parameters were examined for collinearity. Subsequently, two additional univariate binary logistic regression models were performed for each age group, with the presence of DCD as dependent variable and either the calculated variable “movement-quality concerns” or the AIMS scores as independent variables. Alpha was set at 0.05. Finally, correlations between MABC-2 scores after the age of three and AIMS scores and “movement-quality concerns” before the age of three were calculated. 

## 3. Results

### 3.1. Descriptive Results

Results are based on 1507 assessments from 503 participating children. Out of these 503 children, 35 were assessed four times before the age of three, 158 three times, 110 twice, and 170 children were seen once before the age of three. All children were assessed after the age of three. The mean age at last assessment was 5 years 0 months (SD = 1 year 2 months, range 3 year 0 months–10 years 3 months). Table 1 offers an overview of the number of assessments per age group, the corresponding numbers of assessments of children prematurely born, and the number of children with a subsequent (at-risk) DCD diagnosis.

After the age of three, 246 of the 503 (48.9%) participants received a diagnosis of DCD (confirmed diagnoses *n* = 157; at-risk diagnoses *n* = 89). The prevalence of DCD (confirmed or at risk) in the prematurely born group was 118/254 (46.5%). MABC-2 and PDMS-2 scores at the latest assessment are reported in Table 2.

The ratio of boys/girls in the total sample was 1.8/1. This ratio was different in the DCD group (2.6/1) compared to the non-DCD group (1.1/1) and in the group prematurely born (1.3/1) versus term-born (2.5/1) children. The highest ratio was found in the group of term-born children with DCD (4.3/1). 

**Figure 2:**
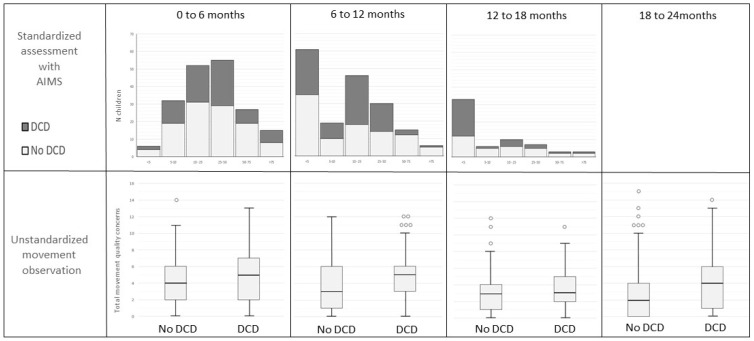
Prevalence of DCD diagnoses after 3 years in the different age groups in relation to percentile ranges of AIMS scores and to the number of movement quality concerns.

The percentage of AIMS scores below the 50th percentile was 77.5% for the assessments at 0–6 months, 88.1% at 6–12 months, and 90.3% at the 12–18-months age group. Figure 2 depicts the prevalence of a subsequent DCD diagnosis in relation to the AIMS-scores and the total number of movement-quality concerns in the different age groups. 

Thirteen categories of movement-quality descriptions were identified: problems concerning hypotonia, hypertonia, laxity, force, asymmetry, control, regulation, organization, coordination, balance, soft neurological signs, dissociation, and planning (comprehensive list in Supplementary Materials Table S2). A category, i.e., “instability”, was not retained in the analyses because no consensus was reached on the content of the terms. The distribution of the 13 movement-quality categories by age and DCD group are reported in Table 3.

### 3.2. Regression Analyses by Age Group

The results of the binary multivariate and the univariate logistic regression analyses are reported in Table 4. The Nagelkerke R square of the models were, respectively, 0.19 for 0–6 months; 0.17 for 6–12 months; 0.08 for 12–18 months; and 0.18 for 18 months–3 years.

A strong association was identified between DCD and male sex irrespective of age group. Prematurity was not significantly associated with a DCD diagnosis. Asymmetry was significantly associated with DCD in the age group 0–6 months. Hypotonia was significantly associated with DCD in the age group 6–12 months. None of the deviant movement-quality categories was significantly associated with DCD in the age group 12–18 months. Finally, hypotonia and poor motor control were associated with DCD in the age group 18 months–3 years. No significant association was obtained between DCD and AIMS scores even when AIMS scores were dichotomized as below or above 10th Pc. DCD was significantly associated with “movement-quality concerns” in all age groups except 12–18 months.

### 3.3. Association between Test Scores 

Significant but weak Pearson correlation coefficient were obtained between the variable “movement-quality concerns” and MABC-2 scores in the age group 0–6 months (rp = −0.23; *p* < 0.001) and 18 months to 3 years (rp = −0.26; *p* = <0.001) albeit not in the age groups 6–12 months (rp = −0.10; *p* = 0.147) and 12–18 months (rp = −0.17; *p* = 0.098). The Spearman correlation coefficients between the AIMS categories and the MABC-2 scores were significant at 6–12 months (rs = 0.162; *p* = 0.037) and 12–18 months (rs= 0.465; *p* < 0.001) but not at 0–6 months (rs = 0.141; *p* = 0.127).

## 4. Discussion

At-risk diagnoses of DCD before the age of three are uncommon in clinical practice. However, follow-up studies of mostly prematurely born children suggest that not only CP but also DCD may be associated with poor motor performance already at a very young age [7,13,15,16]. In this exploratory study in a mixed group of children with developmental concerns, poor movement quality turned out to be manifest before age three in most children later diagnosed with (at-risk) DCD. However, the range of motor performance of the children in this study was small, as most children presented with rather poor motor skills scoring below the mean of the standardization group on the MABC-2. Gross motor dysfunction is associated with several neurodevelopmental disorders involving deficits in cognition, including ASD [28], ADHD [29], and language disorder [30]. This narrow range makes it more difficult to associate DCD with poor movement quality at infant or toddler age and resulted in low R^2^ values of the regression models. Moreover, some children in the non-DCD group in this study may still have been diagnosed with DCD at a later stage, as the mean age at the last assessment was five years.

The moderate association between AIMS scores at 12–18 months and MABC-2 scores after the age of three confirms, to some extent, the predictive validity of the AIMS on a later motor test, as previously reported by Spittle and colleagues [14]. On the other hand, the lack of association in the other age bands is consistent with the findings of Howe and colleagues, who could not establish an association between AIMS scores and MABC-2 scores at the age of 5 years [31]. The binary logistic regression model yielded no significant results for the association between AIMS scores and a DCD diagnosis after the age of three, confirming the results of Prins and colleagues [32]. In contrast, the number of movement-quality concerns expressed by clinicians in reports of non-standardized observations was associated with a DCD diagnosis with an exception in the age group 12–18 months. Confirmed by a significant association between “movement quality-concerns” at 0–6 months and 18 months–3 years and MABC-2-scores, it can be concluded that, already from a very young age, poor movement quality may be observed in children with DCD. The lack of significant prediction at 12–18 months could be related to a lack of power, as the smallest number of assessments were performed in this age group. In addition, in this age group, the transition from crawling to walking may interfere with the observation of movement quality. It is remarkable that it is precisely in this group that the AIMS score was the most predictive of a later DCD diagnosis. This demonstrates how the combination of the AIMS with non-standardized movement observation may be complementary at achieving prediction in different age categories.

The results of the multivariate regression analysis of the different categories of movement-quality concerns need to be interpreted with caution. Firstly, the models could not be validated due to insufficient number of participants. Secondly, the data are based on assessment reports of a limited number of experienced neuro-pediatricians and physiotherapists of only one center. Thirdly, the descriptive terms of the movement-quality observations used are not standardized, and the categorization has not been validated. As such, it is not possible to generalize the results. Nevertheless, the terms and categories could be useful in future studies to develop a standardized observation instrument to evaluate movement quality.

The observations of hypo- and hypertonia are interesting. Clinicians qualified a high number of children in this cohort as hypotonic regardless of a later diagnosis of DCD. The slightly higher number of children with DCD that were considered hypotonic confirm previous research [33,34]. However, hypertonia was also associated with DCD. “Hypertonia” is not a neurological finding in this population but rather may “appear” to be hypertonia because the children are fixing their joints secondary to hypotonia. This is in line with the study of Missiuna and colleagues describing that children with DCD tend to fix or freeze joints during tasks [35]. A recent review on ball catching in children with DCD also described this behavior of fixing joints [36]. It seems plausible that tonus regulation problems, manifesting either as hypo- or hypertonia, are an early indicator of DCD although specificity will probably be low. In line with hypotonia, laxity and weak muscle strength were equally well described in the DCD and non-DCD group. As children with CP were excluded, asymmetry was often seen as asymmetry of the head and/or plagiocephaly, which is also related to hypotonia and as such also had similar prevalence in both groups.

The descriptions used in the categories motor control, regulation, organization, and coordination are partly related to the age group and partly to the jargon used by a specific clinician. For example, poor motor control (mainly described as poor head or trunk control) is mainly used for infants between 0–12 months. Older children all have the basic head and trunk control. Minor motor control problems in later life are rather described as problems with postural control or poor balance reactions. On the other hand, balance problems are not mentioned in the reports of infants between 0–6 months. Neither is there any mention of poor motor planning at an early age. This underlines the need for better defined qualitative descriptions of motor performance by age category.

Soft signs were also mentioned. One may wonder whether these should be regarded as a quality of movement. However, these soft signs, mainly tremor, were rare in this group and do not seem to be specific to the development of DCD. In addition, it was also interesting that the regression models consistently identified sex as a predictive factor even in this already sex-biased group. The important difference in sex ratio in the prematurely born group versus the term-born group with DCD may suggest a different pathogenesis in both groups.

We know from literature that children born very preterm (<32 weeks gestational age) and with very low birthweight (<1500 g at birth) are over six times more likely to develop DCD by school age compared with a control group [12]. However, prematurity was not identified as a predictive factor. The lack of association between DCD and prematurity in this study could be explained by the fact that the complete group needs to be considered at high risk for DCD.

Despite the previously mentioned limitations, this is the first study describing poor movement quality in infants and toddlers with DCD in a mixed cohort of at-risk children. Another advantage of this study is that the DCD diagnosis is the result of a comprehensive multidisciplinary assessment, in which the four DSM-5 criteria are taken into account by an experienced multidisciplinary team, with clinicians interpreting the MABC-2 scores. Low MABC-2 scores do not necessarily indicate DCD, especially at a young age. Children who are tired, lack motivation, or do not understand the task may score low on a test. If the test results are not confirmed by the child’s history and observations, they will not lead to a diagnosis of DCD. On the other hand, especially highly gifted children perform well on the MABC-2, while in everyday life, in complex situations, they still show severe motor problems. These children can still be diagnosed with DCD despite a MABC score above Pc16.

Demonstrating an association between early qualitative motor abnormalities and a later DCD diagnosis was complicated in this study by (1) the fact that a proportion of the children who did not yet receive a DCD diagnosis could still be diagnosed later and (2) that most children without a DCD diagnosis were diagnosed with other neurodevelopmental disorders, such as ADHD, ASD, or learning disabilities. We know that problems in motor development can also be a sign of these other disorders. This also makes it more difficult to distinguish between the DCD and non-DCD groups. Based on the limited associations found, it can therefore be concluded that at least some children with DCD show signs of atypical motor development at an early age.

Further operationalization of qualitative terms may be a way forward to identify the most sensitive and specific features of movement quality in infants and toddlers with DCD. Multicenter prospective longitudinal research with a control group is necessary to develop predictive models supporting clinicians in early identification of children at risk for DCD. Models should not only predict DCD based on one assessment but should also consider the developmental course. As parents report greater delays and difficulties in obtaining a DCD diagnosis, contributing to higher reported stress, this is of high clinical relevance [37]. In addition, further research on the importance of early diagnosis of DCD for treatment and/or counseling of parents is urgently needed. Meanwhile, it seems prudent to counsel parents very thoughtfully, without labeling children too soon and maintaining an open mind about variation in patterns of early development but recognizing that abnormal movement quality in infants can be an early sign of DCD.

## Figures and Tables

**Table 1:** Number of assessed children by age group, gender, corresponding number of children born at preterm age, and number of children with a subsequent clinical DCD or at-risk DCD diagnosis after three years.

Age Category	*n*	Girls *n* (%)	Boys *n* (%)	Preterm Born *n*(%)	DCD or at Risk *n* (%)
0–6 m	223	97 (44)	126 (57)	178 (80)	91 (41)
6–12 m	221	91 (41)	130 (59)	168 (76)	105 (48)
12–18 m	111	44 (40)	67 (60)	67 (60)	59 (53)
18 m–3 y	449	152 (34)	297 (66)	223 * (50)	226 (50)
>3 y	503	181 (36)	322 (64)	254 * (51)	246 (49)

m, months; y, years. * Information on gestational age is missing for 2 adopted children.

**Table 2:** MABC-2 standard scores (mean norm score is 10, SD = 3) or PDMS-2 scores at the last diagnostic assessment of the group with a clinical DCD, at-risk DCD, or no DCD diagnosis.

	MABC-2				PDMS-2 GMQ				PDMS-2 FMQ			
Group	*n*	Mean	SD	Range	*n*	Mean	SD	Range	*n*	Mean	SD	Range
Complete	484	6.18	3.4	1–15	19	77.0	7.9	68–94	19	89.5	13.3	73–118
Risk DCD	86	5.85	2.73	1–15	3	76.3	2.5	74–79	3	95.3	17.2	83–115
DCD	156	3.90	2.21	1–10	1	68	-	-	1	76	-	-
No DCD	242	7.84	3.49	1–15	15	77.7	8.6	68–94	15	89.2	12.8	73–118

MABC-2, Movement Assessment Battery for Children—2; PDMS-2, Peabody Developmental Motor Scales—2; GMQ, Gross Motor Quotient; FMQ, Fine Motor Quotient.

**Table 3:** Frequency of Categories of Qualitative Descriptions in the Reports of the Children with (at-risk) DCD and without DCD.

Category of Terms	0–6 m *n* = 223		6–12 m *n* = 221		12–18 m *n* = 111		18 m–3 y *n* = 449	
	DCD *n* = 91	Non DCD *n* = 132	DCD *n*= 105	Non DCD *n* = 116	DCD *n*= 59	Non DCD *n* = 52	DCD *n* = 226	Non DCD *n* = 223
	*n* (%)	*n* (%)	*n* (%)	*n*(%)	*n* (%)	*n* (%)	*n* (%)	*n* (%)
Hypotonia	34 (37)	54 (41)	70 (67) *	60 (52)	40 (68)	35 (67)	136 (60) *	88 (40)
Hypertonia	68 (75) *	78 (59)	47 (45)	43 (37)	15 (25)	10 (19)	49 (22) *	29 (13)
Laxity	12 (13)	15 (11)	39 (37)	46 (40)	32 (54)	21 (40)	97 (43)	86 (39)
Poor muscle force	9 (10)	10 (8)	3 (3)	0	4 (7)	2 (4)	13 (6)	13 (6)
Asymmetry	57 (63) *	59 (45)	29 (28)	18 (16)	5 (9)	11 (21) *	30 (13)	14 (6)
Poor motor control	27 (30)	36 (27)	28 (27) *	16 (14)	7 (12)	4 (8)	22 (10) *	6 (3)
Poor motor regulation	10 (11)	13 (10)	16 (15)	17 (15)	6 (10)	6 (12)	17 (8)	7 (3)
Poor motor organization	28 (31)	32 (24)	28 (27)	33 (28)	7 (12)	5 (10)	21 (9)	11 (5)
Poor motor coordination	13 (14)	13 (10)	13 (12)	19 (16)	10 (17)	6 (7)	108 (48) *	67 (30)
Poor balance	0	0	12 (11)	8 (7)	10 (17)	11 (21)	95 (42) *	51 (23)
Soft signs	5 (6)	6 (5)	4 (4)	6 (5)	5 (9)	3 (6)	18 (8)	20 (9)
Poor motor dissociation	19 (21) *	15 (11)	24 (23)	16 (14)	7 (12)	4 (8)	12 (5)	6 (3)
Poor motor planning	0	0	1 (1)	2 (2)	3 (5)	1 (2)	23 (10)	15 (7)

m, months; y, years. * These terms were withheld in the regression model.

**Table 4:** Results of Multiple Binary Logistic Regression Analyses to Associate Deviant Movement-Quality Categories (<3 years), Prematurity and Sex with a DCD Diagnosis (>3 years), and Unadjusted Binary Logistic Regression Models to Associate AIMS Scores (<3 years) and “Movement-Quality Concerns” (<3 years) with a DCD Diagnosis (>3 years). Only results with a significance level < 0.10 are reported. Results with a significance level <0.05 are in bold.

Assessments between 0–6 Months (*n* = 223)			
Multiple Regression Model			
Factor	Sig.	OR	95% CI
Sex	<0.001	3.27	1.78–6.01
Prematurity	0.007	2.95	1.34–6.49
Poor motor dissociation	0.097	1.96	0.89–4.34
Asymmetry	0.047	1.81	1.01–3.28
Hypertonia	0.097	1.71	0.91–3.20
Unadjusted Model			
AIMS scores	0.933	1.01	0.80–1.27
Unadjusted Model			
Movement-quality concerns	0.010	1.12	1.03–1.22
Assessments between 6–12 months (*n* = 221)			
Multiple Regression Model			
Sex	<0.001	3.43	1.90–6.19
Poor motor control	0.054	2.04	0.99–4.02
Hypotonia	0.022	1.97	1.10–3.54
Poor motor dissociation	0.083	1.92	0.09–4.02
Unadjusted Model			
AIMS scores	0.510	0.93	0.76–1.14
Unadjusted Model			
Movement-quality concerns	0.041	1.10	1.00–1.20
Assessments between 12–18 months (*n* = 111)			
Multiple Regression Model			
Sex	0.012	2.81	1.26–6.25
Asymmetry	0.053	0.32	0.10–1.0
Unadjusted Model			
AIMS scores	0.056	0.70	0.49–1.01
Unadjusted Model			
Movement-quality concerns	0.659	1.03	0.09–1.18
Assessments between 18 months–3 years (*n* = 449)			
Multiple Regression Model			
Sex	<0.001	3.00	1.94–4.64
Hypertonia	0.069	1.68	0.96–2.93
Poor motor coordination	0.057	1.57	0.99–2.50
Poor balance	0.092	1.53	0.93–2.49
Hypotonia	0.005	1.82	1.19–2.77
Poor motor control	0.050	2.70	1.00–7.30
Unadjusted Model			
Movement-quality concerns	<0.001	1.15	1.08–1.23

Sig., level of significance; OR, odds ratio; CI, confidence interval.

## Data Availability

Data are available from: hilde.vanwaelvelde@ugent.be.

## Supplementary Materials

The following supporting information can be downloaded at: https://www.mdpi.com/article/10.3390/children9030334/s1, Table S1: List of excluded diagnoses; Table S2: Frequency of movement-quality descriptions in the different age groups.
