# Supplementary material for: Developmental Coordination Disorder before the Age of Three: A Longitudinal Retrospective Study in a Belgian Center for Developmental Disabilities
Source: Children (Basel). 2022 Mar 2;9(3):334. doi: 10.3390/children9030334 (PMC8947167; doi:10.3390/children9030334)
Supplement: Supplementary file 1 [file children-09-00334-s001.zip › children-1540215-supplementary.pdf]

children

Table S1. List of excluded diagnoses.

Central neurological disorder	Cerebral palsy
Skeletal dysfunctions	Arthrogryposis<br>Extreme skeletal problems
Peripheral neurological disorders	Obstetric Brachial Plexus Lesion
Specific syndromes	Aarskog syndrome<br>Angelman syndrome<br>Beals syndrome<br>Charge syndrome<br>Down syndrome<br>Fragile X syndrome<br>Goldenhar syndrome<br>Gorlin syndrome<br>Joubert syndrome<br>Klinefelter syndrome<br>Moebius syndrome<br>Poland syndrome<br>Prader-Willi syndrome<br>Williams- Beuren syndrome
Neuromuscular diseases	Muscular dystrophy (Becker muscular dystrophy, Duchenne, Strümpell-lorrain disease)<br>Myopathy
Spinal cord abnormalities	Chiari malformation<br>Meningomyelocele<br>Spina bifida

Table S2. Frequency of movement-quality descriptions in the different age groups

**Age group**	**0-6mo**	**6-12mo**	**12-18mo**	**18mo-3y**
Number of children	223	221	111	449
**Hypotonia**				
General Hypotonia	27	74	49	135
Mild hypotonia	30	43	21	82
Severe hypotonia	0	1	4	2
Proximal Hypotonia	72	87	28	84
Distal Hypotonia	10	11	6	13
Hypotonia face	3	2	5	24
Problems moving against gravity	10	4	0	0
**Hypertonia**				
General hypertonia	126	59	18	41
Mild hypertonia	36	11	1	13
Severe hypertonia	1	0	0	0
Proximal hypertonia	10	5	3	1
Distal hypertonia	94	57	12	59
**Laxity**				
General laxity	6	50	35	127
Laxity lower limbs	26	54	33	100
Laxity upper limbs	0	1	3	9
**Poor muscle force**				
General force problem	4	3	4	18
Force problems lower limbs	0	0	2	1
Force problems upper limbs	1	0	1	1
Force problems of the abdominal muscles	14	0	0	7
**Asymmetry**				
General asymmetry	33	14	9	11
Asymmetry lower limbs	32	22	7	29
Asymmetry upper limbs	32	12	3	4
Asymmetry of the head	90	11	5	9
Asymmetry of the trunk	2	4	0	0
Plagiocephaly	36	17	6	8
**Poor motor control**				
General control problems	23	19	3	12
Problems with trunc control	47	32	10	17
Problems with head control	36	6	0	0
**Poor motor regulation**				
Problems with Gradation of movements	4	21	9	13

Overshooting	1	1	1	1
Tone regulation problems	20	19	4	13
**Poor motor organization**				
Problems with postural changes	4	50	8	15
Problems of movement organization on the midline	38	2	0	0
Problems in organizing movements	24	8	2	15
Problems in organizing weight shift	8	23	6	12
Chaotic movements	8	4	0	0
**Poor motor coordination**				
Mild coordination problems	1	0	1	27
Moderate coordination problems	0	1	0	1
General coordination problems	11	16	6	91
Stiff movements	12	16	12	115
Clumsy movements	0	0	0	3
Awkward movements	0	0	0	2
Jerky	0	0	0	2
Shaky movements	6	1	0	1
Disturbed coordination	0	0	1	2
Disturbed mimanual coordination	0	1	0	34
Use of dystonic like movements	6	5	1	1
Use of diskenetic like movement	0	1	0	1
Disturbed diadochokinesis	0	0	0	4
**Poor balance**				
Mild Balance Problems	0	0	2	27
Moderate balance problems	0	0	0	0
Severe balance problems	0	1	0	1
Wider base of support	0	0	12	58
Atactic like movements	0	2	1	5
Drunken appearing gait	0	0	0	1
Stumbling	0	5	7	40
Poor balance reactions	0	5	2	6
Poor balance	0	5	5	75
Slower supporting reactions	0	5	0	2
Slower balance reactions	0	1	1	0
**Soft signs**				
Tremor	7	9	7	28
Dysmetria	0	2	1	8
Clonus	3	1	1	4
Overflow upper limbs	1	0	0	0
**Poor motor dissociation**				
Stereotyped movements	7	4	0	4

Less dissociated movements	3	2	0	0
Less selectivity	8	5	0	2
Dissociating problems	21	33	11	12
**Poor motor planning**				
Global planning problems	0	0	1	16
Action planning problems	0	3	0	18
Poor motor planning	0	1	3	18
